# Prediction of MYCN Amplification, 1p and 11q Aberrations in Pediatric Neuroblastoma *via* Pre-therapy 18F-FDG PET/CT Radiomics

**DOI:** 10.3389/fmed.2022.840777

**Published:** 2022-03-18

**Authors:** Luodan Qian, Shen Yang, Shuxin Zhang, Hong Qin, Wei Wang, Ying Kan, Lei Liu, Jixia Li, Hui Zhang, Jigang Yang

**Affiliations:** ^1^Department of Nuclear Medicine, Beijing Friendship Hospital, Capital Medical University, Beijing, China; ^2^Department of Surgical Oncology, National Center for Children's Health, Beijing Children's Hospital, Capital Medical University, Beijing, China; ^3^Sinounion Medical Technology (Beijing) Co., Ltd., Beijing, China; ^4^Department of Molecular Medicine and Pathology, School of Medical Science, The University of Auckland, Auckland, New Zealand; ^5^Department of Laboratory Medicine of Medical School, Foshan University, Foshan, China; ^6^Department of Biomedical Engineering, School of Medicine, Tsinghua University, Beijing, China

**Keywords:** 18F-FDG PET/CT, radiomics, neuroblastoma, MYCN amplification, 1p aberration, 11q aberration

## Abstract

**Purpose:**

This study aimed to assess the predictive ability of 18F-FDG PET/CT radiomic features for MYCN, 1p and 11q abnormalities in NB.

**Method:**

One hundred and twenty-two pediatric patients (median age 3. 2 years, range, 0.2–9.8 years) with NB were retrospectively enrolled. Significant features by multivariable logistic regression were retained to establish a clinical model (C_model), which included clinical characteristics. 18F-FDG PET/CT radiomic features were extracted by Computational Environment for Radiological Research. The least absolute shrinkage and selection operator (LASSO) regression was used to select radiomic features and build models (R-model). The predictive performance of models constructed by clinical characteristic (C_model), radiomic signature (R_model), and their combinations (CR_model) were compared using receiver operating curves (ROCs). Nomograms based on the radiomic score (rad-score) and clinical parameters were developed.

**Results:**

The patients were classified into a training set (*n* = 86) and a test set (*n* = 36). Accordingly, 6, 8, and 7 radiomic features were selected to establish R_models for predicting MYCN, 1p and 11q status. The R_models showed a strong power for identifying these aberrations, with area under ROC curves (AUCs) of 0.96, 0.89, and 0.89 in the training set and 0.92, 0.85, and 0.84 in the test set. When combining clinical characteristics and radiomic signature, the AUCs increased to 0.98, 0.91, and 0.93 in the training set and 0.96, 0.88, and 0.89 in the test set. The CR_models had the greatest performance for MYCN, 1p and 11q predictions (*P* < 0.05).

**Conclusions:**

The pre-therapy 18F-FDG PET/CT radiomics is able to predict MYCN amplification and 1p and 11 aberrations in pediatric NB, thus aiding tumor stage, risk stratification and disease management in the clinical practice.

## Introduction

Neuroblastoma (NB), the most common extracranial solid pediatric tumor, accounts for about 8–10% of all childhood cancer and 12–15% of childhood cancer mortality (1). Using selected clinical, pathologic, and genetic factors, patients diagnosed with NB can be classified into different risk groups for treatment (2). Previous studies have shown that patient outcomes of NB are highly correlated with risk stratification, with more than 90% cure in non-high risk patients and <50% event-free survival rate in high risk patients (3). It is therefore very important to obtain a better understanding of risk factors so that treatment strategies for children with NB can be tailored accordingly. Previous studies have demonstrated the value of prognostic factors such as patients age, tumor stage using the International Neuroblastoma Staging System (INSS), tumor histopathology using the International Neuroblastoma Pathology Classification (INPC) system, DNA ploidy, cytogenetics such as MYCN amplification status and chromosome aberrations of 1p and 11q (1, 4, 5). In addition, CT or MR image-defined risk factors (IDRFs) were used to distinguish low-risk tumors from high-risk tumors (6, 7). However, the predictive value of nuclear medicine functional imaging techniques on tumor biology has been less studied.

Nuclear medicine functional imaging plays an important role in the assessment of NB. Currently, ^123^I-Metaiodobenzylguanidine (^123^I-MIBG) scintigraphy is a standard practice in the diagnosis of NB (6), with ~90% of patients having MIBG avid tumors. However, in some countries, including China, ^123^I-MIBG has not been approved for clinical use and cannot be included in the standard clinical protocols for NB patients. In our practice, we have utilized ^18^F-fluorodeoxyglucose positron emission tomography/computer tomography (^18^F-FDG PET/CT) in the diagnosis and follow-up of NB patients. ^18^F-FDG PET imaging has been reported to be equal or superior to ^123^I-MIBG scan for delineating NB disease extent in the chest, abdomen, and pelvis (8). In case the tumor is not MIBG avid, ^18^F-FDG PET is also recommended as a complementary option to ^123^I-MIBG scintigraphy (9).

The purpose of this study aims to evaluate whether diagnostic ^18^F-FDG PET/CT imaging plays a role in risk stratification prediction in children with NB. The relationship between diagnostic ^18^F-FDG PET/CT image features and the tumor biology of NB were investigated to answer this question. Specifically, cytogenetic factors, MYCN amplification status and chromosome aberrations of 1p and 11q, are chosen as representative indicators of tumor biology. It was well-documented that MYCN amplification and chromosome aberrations of 1p and 11q are powerful prognostic markers and have a strong association with worse outcome in NB (5). Amplification of MYCN can be detected in 20% of cases with NB and is closely linked with high-risk disease and poorer outcome (10). Loss of heterozygosity on chromosome 1p and 11q are correlated with increased disease severity (2, 11). For the PET/CT image analysis method, radiomic analysis was chosen in this study. In contrast to conventional visual image features, radiomics is expected to provide more comprehensive description of tissues, with the potential to aid clinical care in several aspects including diagnosis, prognosis and treatment selection (12, 13). Currently, a number of studies demonstrated the value of ^18^F-FDG PET/CT-based radiomics in predicting the histological subtypes of lung cancer (14) and distinguishing breast carcinoma from breast lymphoma (15). So far, there is little study to investigate the predictive value of ^18^F-FDG PET/CT on the status of MYCN, 1p and 11q in pediatric NB. Therefore, this study was designed to evaluate whether ^18^F-FDG PET/CT-based radiomics can predict the status of MYCN, 1p and 11q, which in turn, can be used in risk stratification prediction in children with NB.

## Methods

### Patients

The records of 139 pediatric patients with newly diagnosed NB were reviewed retrospectively between March 2018 and November 2019 in our hospital. The inclusion criteria were as follows: (1) pathologically confirmed NB; (2) age ≤ 18 years at diagnosis; (3) complete PET/CT imaging data; (4) complete clinical information; (5) no cancer therapy before PET/CT imaging; (6) complete MYCN amplification and 1p and 11q aberrations data. Subsequently, 17 cases were excluded because of unavailable MYCN, 1p and 11q information, and 122 patients were included in this study. These patients were randomly divided into training set and test set with a ratio of 7:3. This retrospective study was approved by Institutional Review Board of our hospital and the requirement of written informed consent was waived.

### Determination of MYCN Amplification and 1p and 11q Aberrations by FISH

MYCN amplification and 1p and 11q aberrations were determined using FISH from paraffin-embedded tissue obtained by biopsy or surgery at initial diagnosis according to the previously published method (16). According to the recommendations of the European Neuroblastoma Quality Assessment group (17, 18), MYCN amplification was defined as a > four-fold increase of signals.

### Clinical Data and 18F-FDG PET/CT Imaging

#### Clinical Characteristics

Patient gender, age, neuron-specific enolase (NSE), serum ferritin (SF), lactate dehydrogenase (LDH), vanillylmandelic acid (VMA), homovanillic acid (HVA), maximum tumor diameter (MTD) in Ultrasound, and MTD in CT and/or MRI.

All patients underwent whole body scan on the PET/CT scanner (Biograph mCT-64 PET/CT; Siemens, Knoxville, Tenn) in accordance with EANM guidelines (19, 20) and a biopsy/surgery for pathological diagnosis of NB was performed within 3 months. The PET scan was carried out with 3 min per bed position immediately after the whole body CT scan. PET images were reconstructed using the ordered subsets-expectation maximization algorithm with time-of-flight. The regions-of-interest (ROIs) of primary tumor were manually drawn by an experienced nuclear medicine physician using the longitudinal PET/CT module in 3D Slicer (version 4.10.1). ROIs were delineated along the edge of NB on CT images, which included the entire tumor, metastatic lesions and unclear demarcations between the primary tumor and its surrounding metastasis. In order to map to the PET image, the ROIs were resampled based on B-spline interpolation to ensure that it had the same pixel spacing as the PET image.

### Feature Extraction and Selection and Model Construction

Univariate analysis was performed to compare the differences in clinical characteristics. Based on the selected characteristics, a clinical model (C-model) was established.

**Figure 1:**
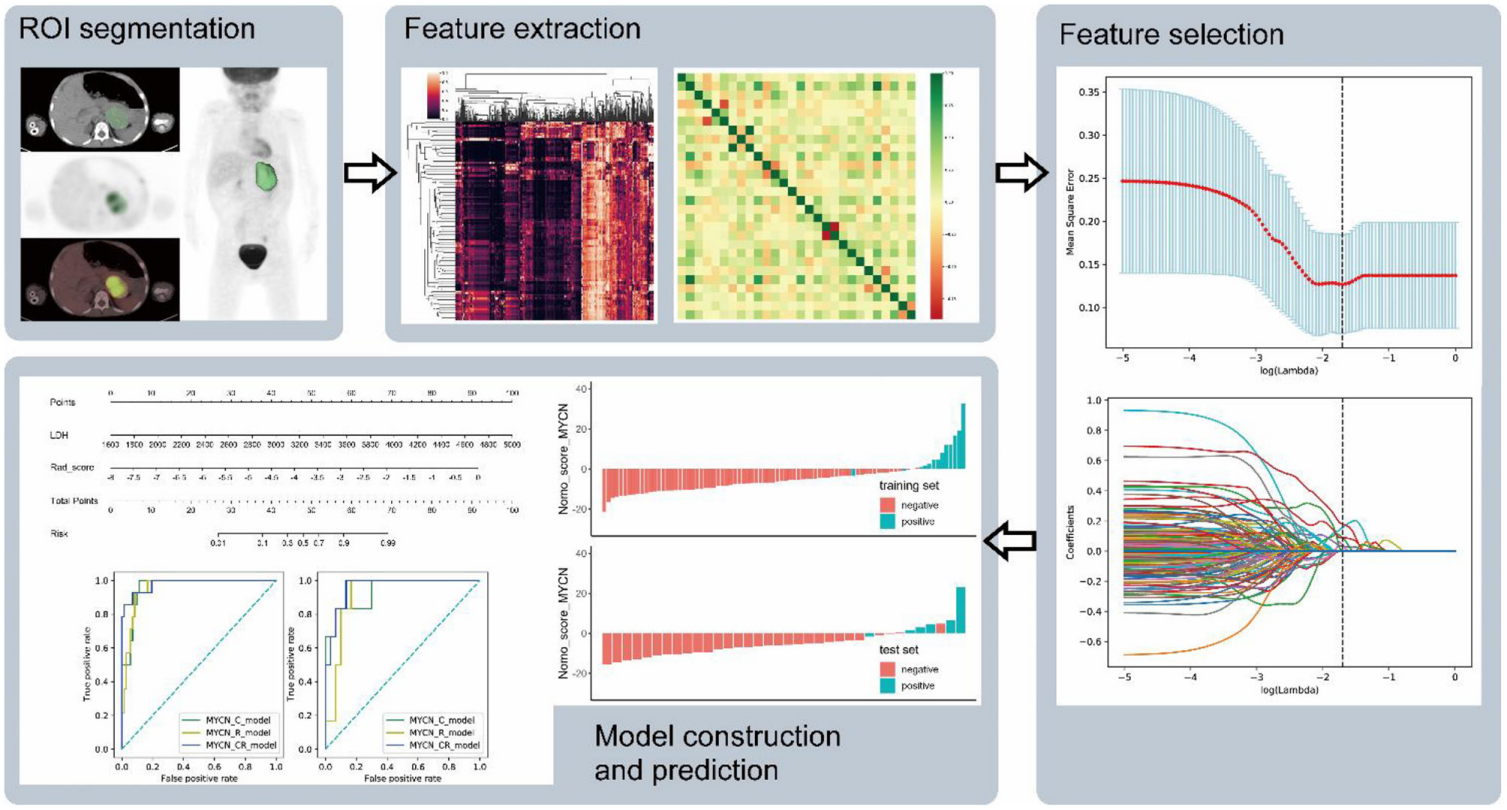
The flow chart shows the process of ROI segment, feature extraction, feature selection, and model construction and prediction.

Radiomic features from CT and PET images were computed separately using pyradiomics, an open-source python package for the extraction of radiomic features from medical imaging (21). First order features (*n* = 18), shape features (*n* = 14), gray level co-occurrence matrix (GLCM) features (*n* = 24), gray level run length matrix (GLRLM) features (*n* = 16), gray level size zone matrix (GLSZM) features (*n* = 16), neighboring gray tone difference matrix (NGTDM) features (*n* = 5), and gray level dependence matrix (GLDM) features (*n* = 14) were extracted from the original and the pre-processed images. The following methods were used in the imaging processing: wavelet filtering, square, square root, logarithm, exponential and gradient filtering (Figure 1).

Intraclass correlation coefficients (ICC) were obtained to assess the reliability of variables using the features extracted from the two sets of ROIs portrayed separately by two different nuclear medicine physicians in 24 out of the 122 patients with NB after 2 months. Because of imbalanced datasets, synthetic minority oversampling technique (SMOTE) was used to improve random oversampling in the training set. Least absolute shrinkage and selection operator (LASSO) was applied for variable selection and regularization in the training set. Predictive R_models were built by logistic regression and the radiomic score (rad-score) for each patient was computed based on the selected radiomic features. Additionally, the selected clinical characteristics combined with radiomics features were used to construct the combination model (CR_model). All models were built and trained in the training set, and the prediction performance was evaluated in the training and test sets. Ten-fold cross-validation was applied to prevent model overfitting in the training process. Receiver operating characteristic (ROC) curve and area under curve (AUC) were employed for the evaluation of the diagnostic performance in the training and test sets.

### Statistical Analysis

Statistical analyses were performed with Python (ver. 3.7.8, www.python.org) and R (ver. 4.0.3, www.r-project.org). The Python packages of “sklearn,” “numpy,” and “pandas” were used for LASSO binary logistic regression and ROC curve; the “scipy” was for analyzing statistical properties; the “imblearn” was for SMOTE. The R package “rms” was employed to create nomograms. The *t*-test or Mann-Whitney *U*-test was applied for univariate analysis, and *p* < 0.05 with a 95% confidence interval was considered as statistical significance. AUC-ROC curve was calculated for evaluating the diagnostic performance of models. AUC ranging from 0.5 to 1.0 is commonly used as a measure of classifier performance. A value of 0.5 is equal to random guessing, while 1.0 means a perfect classifier.

## Results

### Clinical Characteristics of Patients

According to the inclusion criteria, 122 out of 139 patients with NB were enrolled in this study. Eighty six patients were assigned to the training set and 36 patients were assigned to the test set. All clinical characteristics are summarized in Table 1, including gender, age, neuron-specific enolase (NSE), serum ferritin (SF), lactate dehydrogenase (LDH), vanillylmandelic acid (VMA), homovanillic acid (HVA), maximum tumor diameter (MTD) in Ultrasound, and MTD in CT and/or MRI. The percentages of MYCN-, 1p- and 11q-positive cases were 16.4% (20/122), 38.5% (47/122), and 39.3% (48/122), respectively. Among these variables, NSE, LDH, VMA, and MTD in CT/MRI were significantly different between MYCN-positive and negative groups (All *p* < 0.05). Between 1p-positive and negative cases, NSE, LDH, VMA, MTD in Ultrasound and MTD in CT/MRI were distinct (All *p* < 0.05). Between 11q-positive and negative cases, age, SF, LDH, VMA, and HVA were distinct (All *p* < 0.05) (Table 1).

**Table 1:** Clinical features of NB patients.

**Clinical features**	**Total**	**MYCN**			**1p**			**11q**		
		**Positive**	**Negative**	***p*** **-value**	**Positive**	**Negative**	***p*** **-value**	**Positive**	**Negative**	***p*** **-value**
Number	122	20	102		47	75		48	74	
Gender				0.224			0.062			0.345
Male	52	11	41		25	27		23	29	
Female	70	9	61		22	48		25	45	
Age (year)	3.2 (0.2–9.8)	2.5	3.4	0.1082	3.4	2.8	0.0885	4.0	2.3	0.0002
NSE (ng/ml)	219.1 (14.7–2627.1)	666.5	152.6	0.0046	370.0	129.1	0.0004	336.2	128.8	0.2977
SF (ng/ml)	210.2 (8.1–1807.0)	216.6	202.0	0.0744	220.1	189.5	0.0929	247.8	117.8	0.0019
LDH (U/L)	553 (177–6029)	2261	427	0.0001	936	386	<0.0001	596	411	0.0460
VMA	236.2 (5.2–5975.0)	28.6	364.8	<0.0001	164.2	396.9	0.0055	507.6	98.3	0.0080
HVA	54.7 (1.5–1532.0)	42.5	69.3	0.1169	51.1	61.8	0.0526	108.6	33.4	0.0141
MTD Ultra (cm)	9.1 (2.2–20.0)	11.3	9.0	0.0820	10.5	8.4	0.0161	9.6	8.7	0.0882
MTD CT/MRI (cm)	9.3 (2.1–17.4)	11.4	9.1	0.0382	11.1	9.0	0.0044	10.1	9.1	0.1196

*Each feature was expressed as median (minimum–maximum) except for gender*.

*NSE, neuron-specific enolase; SF, serum ferritin; LDH, lactate dehydrogenase; VMA, Vanillylmandelic Acid; HVA, homovanillic acid; MTD Ultra, maximum tumor diameter (MTD) in ultrasound; MTD CT/MRI, MTD in CT/MRI*.

### Predictive Model Construction

The total of 2,632 radiomic features were extracted from PET/CT images using pyradiomics. After assessing the robustness, 1,623 out of 2,632 features retained for model building, with intraclass correlation coefficients (ICC) > 0.75. In respect of C-model (clinical variables) constructed by logistic regression and trained in the training set, 4 clinical characteristics (LDH, NSE, VMA, and SF) were selected for MYCN prediction, with 3 characteristics (LDH, NSE and age) for 1p prediction and 3 characteristics (LDH, SF and HVA) for 11q prediction. As for R_model (radiomics signature) establishment, 6 radiomic features were chosen for MYCN prediction, with 8 features for 1p prediction and 7 features for 11q prediction (Table 2 and Supplementary Table 1).

**Table 2:** Comparison of the radiomic features between positive and negative in training sets of R_model.

**Radiomic feature**	***p*** **-value**
**MYCN**	
PET_squareroot_gldm_HighGrayLevelEmphasis	0.0234
PET_wavelet-LHL_gldm_DependenceNonUniformity	0.0233
PET_wavelet-HHH_glszm_SizeZoneNonUniformity	0.0361
CT_logarithm_firstorder_Skewness	0.0001
CT_wavelet-LLL_gldm_DependenceVariance	0.0009
CT_wavelet-HLL_glszm_LargeAreaHighGrayLevelEmphasis	0.0156
**1p**	
PET_squareroot_glcm_Idmn	0.0009
PET_logarithm_firstorder_Minimum	0.0940
PET_wavelet-LLL_glcm_InverseVariance	0.0061
PET_wavelet-HHL_gldm_DependenceVariance	0.0436
PET_wavelet-HHH_glszm_SmallAreaHighGrayLevelEmphasis	<0.0001
PET_wavelet-HHH_glszm_LowGrayLevelZoneEmphasis	0.0002
CT_exponential_glszm_SmallAreaEmphasis	0.0554
CT_wavelet-HHH_glszm_SizeZoneNonUniformityNormalized	0.0885
**11q**	
PET_original_glszm_GrayLevelNonUniformity	0.0108
PET_wavelet-LHL_gldm_DependenceNonUniformityNormalized	0.0271
CT_original_shape_Flatness	0.0043
CT_wavelet-LLL_glrlm_RunVariance	0.0006
CT_wavelet-LHL_firstorder_Median	0.0613
CT_wavelet-LHL_glcm_Imc1	0.0166
CT_wavelet-HHH_firstorder_Entropy	0.0291

In regard to CR_model (combinations of clinical and radiomic features) construction, eight features were chosen for MYCN prediction, which included 4 clinical characteristics (NSE, LDH, VMA, and MTD in CT/MRI) and 2 PET, 2 CT features (Tables 1, 3). Eleven features were selected for 1p prediction, which included 5 clinical characteristics (NSE, LDH, VMA, MTD in Ultrasound and MTD in CT/MRI) and 5 PET, 1 CT features (Tables 1, 3). Eleven features were picked up for 11q prediction, which included 5 clinical characteristics (age, SF, LDH, VMA, and HVA) and 1 PET, 5 CT features (Tables 1, 3).

**Table 3:** Comparison of the radiomic features between positive and negative in training sets of CR_model.

**Radiomic feature**	***p*** **-value**
**MYCN**	
PET_wavelet-LLH_glszm_GrayLevelNonUniformity	0.0125
PET_wavelet-HHH_glszm_SizeZoneNonUniformity	0.0361
CT_exponential_glrlm_LongRunEmphasis	0.0224
CT_wavelet-HHL_firstorder_Maximum	0.0832
**1p**	
PET_squareroot_ngtdm_Contrast	0.0286
PET_logarithm_firstorder_Minimum	0.0940
PET_wavelet-LLH_glrlm_LongRunLowGrayLevelEmphasis	0.0105
PET_wavelet-HHH_glszm_SmallAreaHighGrayLevelEmphasis	<0.0001
PET_wavelet-HHH_glszm_LowGrayLevelZoneEmphasis	0.0002
CT_exponential_glszm_SmallAreaEmphasis	0.0554
**11q**	
PET_wavelet-LHL_gldm_DependenceNonUniformityNormalized	0.0271
CT_wavelet-LLL_glrlm_RunVariance	0.0006
CT_wavelet-LHL_firstorder_Median	0.0613
CT_wavelet-LHL_glcm_Imc1	0.0166
CT_wavelet-HLL_glrlm_LowGrayLevelRunEmphasis	0.0037
CT_wavelet-HHH_firstorder_Entropy	0.0291

Rad-scores were calculated by the following formula:
Rad_score_MYCN = −2.6446
+ 0.17750 × PET_wavelet-LLH_glszm_GrayLevelNonUniformity
+ 0.88251 × PET_wavelet-HHH_glszm_SizeZoneNonUniformity
– 0.00069 × CT_exponential_glrlm_LongRunEmphasis
– 0.02217 × CT_wavelet-HHL_firstorder_Maximum
Rad_score_1p = 2.9612
– 115.24 × PET_squareroot_ngtdm_Contrast
– 0.29673 × PET_logarithm_firstorder_Minimum
+ 0.04218 × PET_wavelet-LLH_glrlm_LongRunLowGrayLevelEmphasis
+ 2.1217 × PET_wavelet-HHH_glszm_SmallAreaHighGrayLevelEmphasis
– 5.5262 × PET_wavelet-HHH_glszm_LowGrayLevelZoneEmphasis
– 5.1213 × CT_exponential_glszm_SmallAreaEmphasis
Rad_score_11q = −2217.3
– 147.63 × PET_wavelet-LHL_gldm_DependenceNonUniformityNormalized
– 0.41560 × CT_wavelet-LLL_glrlm_RunVariance
– 0.59915 × CT_wavelet-LHL_firstorder_Median
+ 58.736 × CT_wavelet-LHL_glcm_Imc1
– 14.536 × CT_wavelet-HLL_glrlm_LowGrayLevelRunEmphasis
+ 2232.9 × CT_wavelet-HHH_firstorder_Entropy.

The *p*-values of radiomic features are shown in Table 3. Rad-scores presented significant difference between positive and negative groups in the training and test sets (*p* < 0.001). NB with MYCN, 1p and 11q positive had higher Rad-score than those with negative in both the training and test sets.

**Figure 2:**
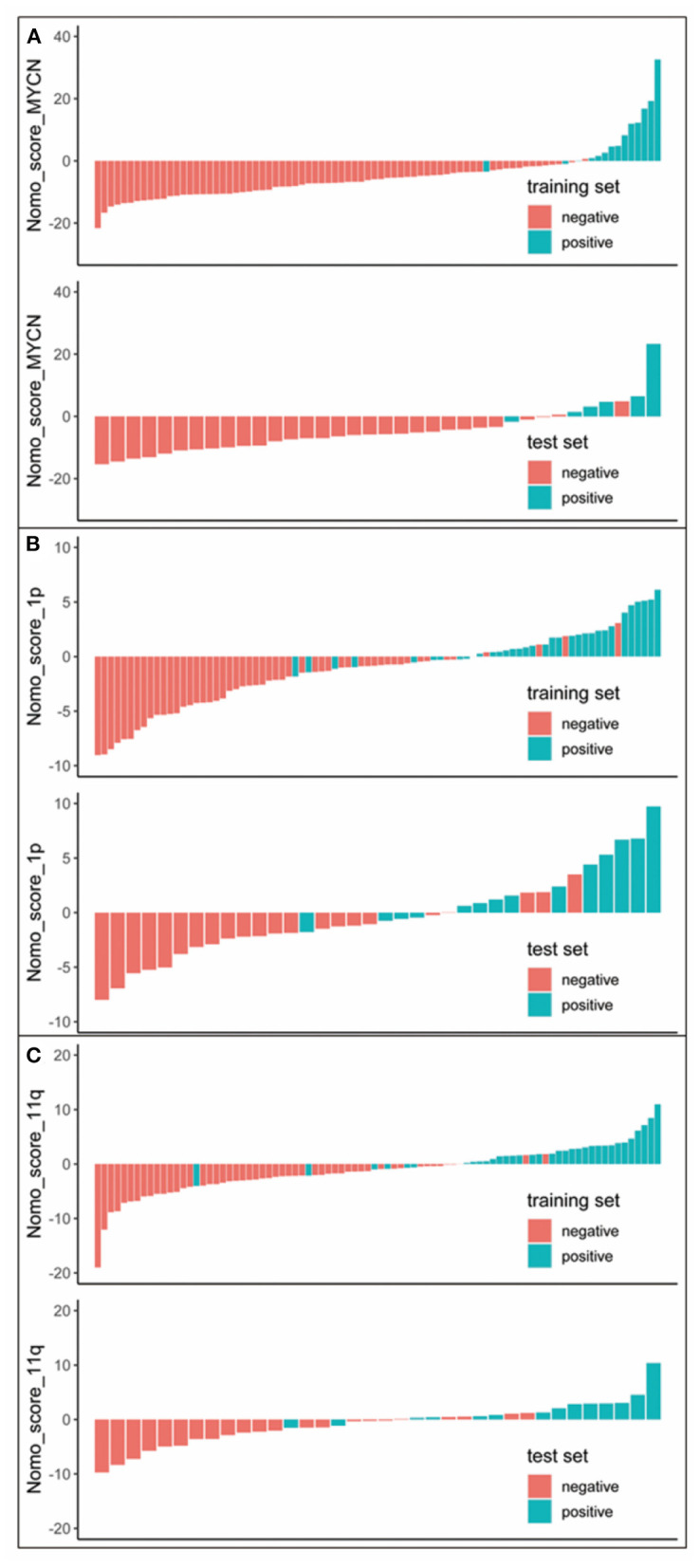
Nomo_score for every patient in each set. The red marks indicate negative samples, while the blue marks indicate the positive samples. **(A)** Nomo_score of MYCN status prediction. **(B)** Nomo_score of 1p status prediction. **(C)** Nomo_score of 11q status prediction.

Nomogram score (Nomo_score) was calculated by the following formula (Figure 2):

Nomo_score_MYCN = −0.7569 + 0.0064 × LDH + 2.4857 × Rad_score_MYCN
Nomo_score_1p = −0.5175 + 0.0017 × LDH + 1.0476 × Rad_score_1p
Nomo_score_11q = −0.3897 – 0.0020 × LDH + 0.0088 × SF + 1.6657 × Rad_score_11q

**Figure 3:**
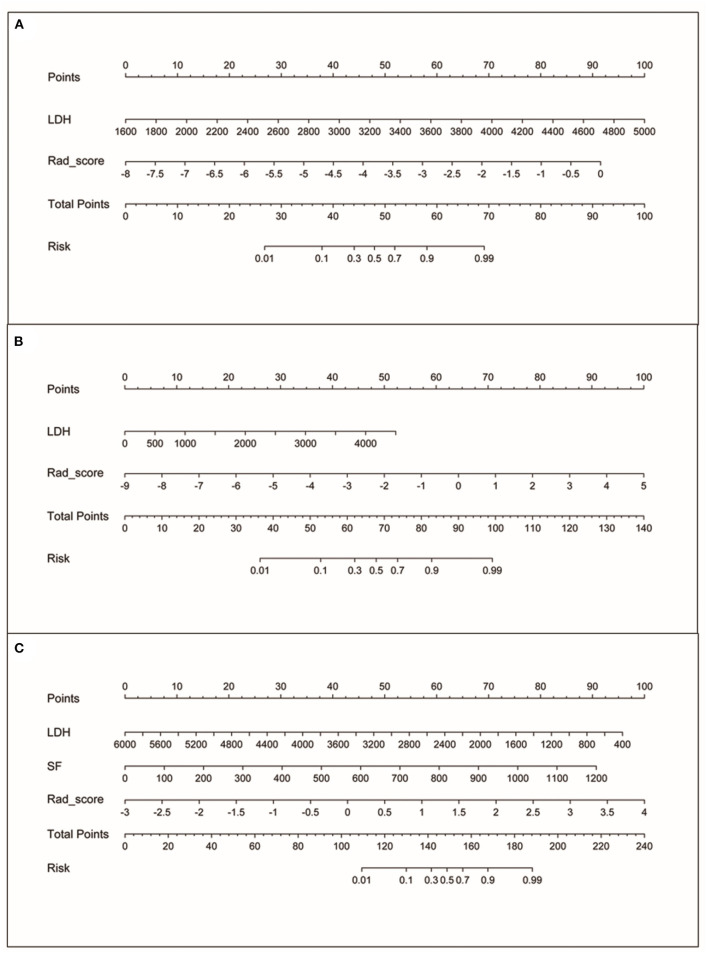
The nomograms. **(A)** Nomogram based on rad-score and LDH for MYCN status prediction. **(B)** Nomogram based on rad-score and LDH for 1p status prediction. **(C)** Nomogram based on rad-score, LDH and SF for 11q status prediction.

The nomogram was created based on the training set, which represented individualized prediction and visualized proportion of each factor (Figure 3).

### Model Performance

**Figure 4:**
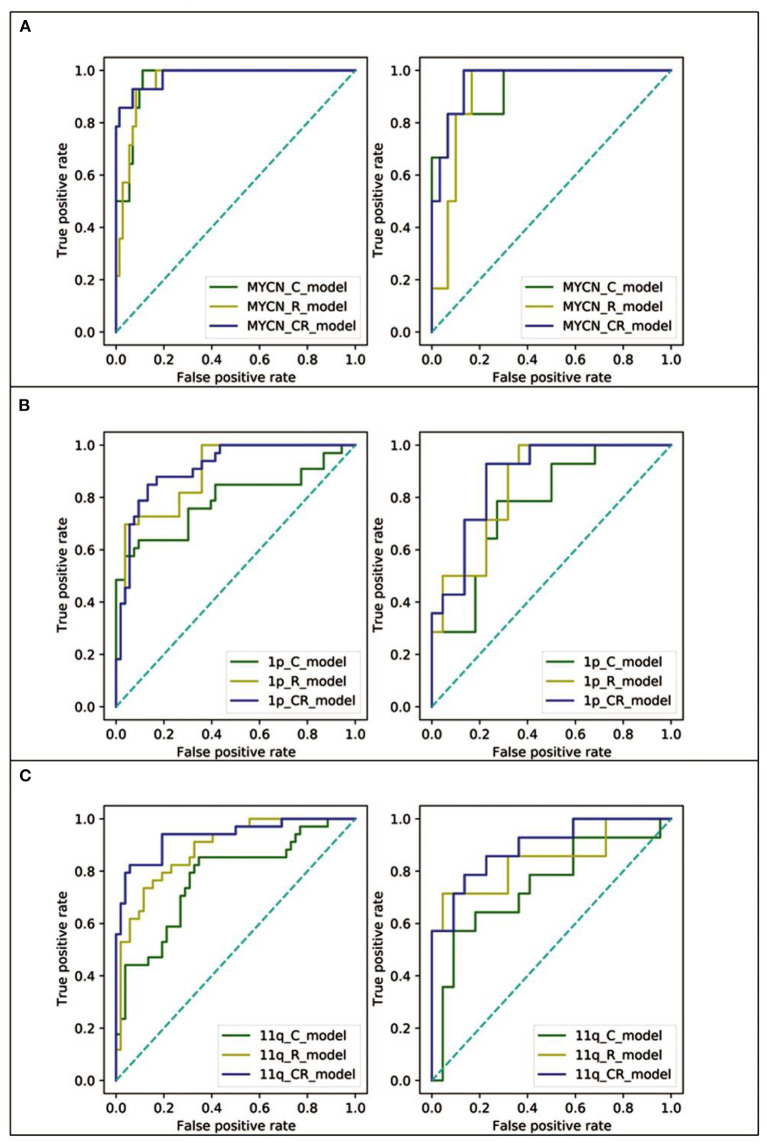
The ROC curves of the C_model (green line), R_model (yellow line), and CR_model (blue line) in both training (left) and test (right) set. **(A)** The ROC curves of MYCN status prediction. **(B)** The ROC curves of 1p status prediction. **(C)** The ROC curves of 11q status prediction.

To evaluate the performance in predicting MYCN, 1p and 11q status, C_model, R_model and CR_model were compared. The predictive abilities of models (sensitivity, specificity, and AUC) were shown in Table 4, and ROC curves were displayed in Figure 4. Obviously, the CR_models were the best predictive models for MYCN, 1p and 11q abnormalities, with AUCs of 0.98 (sensitivity, 0.93; specificity, 0.93), 0.91 (sensitivity, 0.85; specificity, 0.83), and 0.93 (sensitivity, 0.82; specificity, 0.90) in the training set, respectively. In the test set, their AUCs were 0.96 (sensitivity, 0.83; specificity, 0.87), 0.88 (sensitivity, 0.79; specificity, 0.77), and 0.89 (sensitivity, 0.86; specificity, 0.72), sequentially. The CR_model for MYCN prediction had the greatest performance in the training and test sets compared to the CR_models for 1p and 11q prediction. In addition, the R_models for predicting 1p and 11q performed better than the C_models in the test set (AUCs = 0.85 vs. 0.77 for 1p; AUCs = 0.84 vs. 0.74 for 11q). In contrast, the C_model for MYCN prediction was better than the R_model in the test set (AUCs = 0.94 vs. 0.92).

**Table 4:** The predictive value of the models in MYCN, 1p and 11q.

**Model**	**Training set**				**Test set**			
	**Sensitivity**	**Specificity**	**Accuracy**	**AUC (95%CI)**	**Sensitivity**	**Specificity**	**Accuracy**	**AUC (95%CI)**
**MYCN**								
C_model	1.00	0.88	0.90	0.96 (0.93–0.99)	0.83	0.93	0.92	0.94 (0.85–1.00)
R_model	0.86	0.92	0.91	0.96 (0.93–0.99)	0.83	0.90	0.89	0.92 (0.82–1.00)
CR_model	0.93	0.93	0.93	0.98 (0.96–0.99)	0.83	0.87	0.86	0.96 (0.90–1.00)
**1p**								
C_model	0.64	0.71	0.68	0.79 (0.73–0.85)	0.79	0.59	0.67	0.77 (0.62–0.91)
R_model	0.73	0.75	0.74	0.89 (0.85–0.93)	0.93	0.64	0.75	0.85 (0.73–0.97)
CR_model	0.85	0.83	0.84	0.91 (0.87–0.95)	0.79	0.77	0.78	0.88 (0.78–0.98)
**11q**								
C_model	0.71	0.73	0.72	0.77 (0.71–0.83)	0.64	0.64	0.64	0.74 (0.60–0.88)
R_model	0.76	0.83	0.80	0.89 (0.85–0.93)	0.79	0.68	0.72	0.84 (0.73–0.95)
CR_model	0.82	0.90	0.87	0.93 (0.90–0.96)	0.86	0.72	0.77	0.89 (0.79–0.99)

## Discussion

Considering the well-established role of MYCN, 1p and 11q abnormalities in the prognosis of NB, identifying these events are crucial for risk stratification. This study provided three distinct forms of predictive models (clinical variables, radiomic signature and their combinations) for identifying MYCN and chromosomal abnormalities in a non-invasive way, demonstrating that pre-therapy ^18^F-FDG PET/CT-based radiomics had an extremely important role in predicting MYCN amplification and 1p and 11q aberrations. In particular, CR_model was suggested to be the best model for the prediction of MYCN, 1p and 11q status with the largest AUCs in the training and test sets.

Recently, clinical variables (such as LDH and SF) have been demonstrated to be prognostic biomarkers in large-scale studies, which suggested to reconsider utilizing LDH and SF as NB risk stratification factors (22, 23). In the present study, LDH and SF were also predictors of MYCN, 1p and 11q abnormalities. The radiomics models had a power to predict these aberrations, but models integrating PET and CT features with clinical variables led to higher predictive performance for training and test cohorts, in comparison with models with radiomic features or clinical parameters alone (Table 2). In line with other studies (24), the integration of radiomic features with clinical parameters has a complementary and added impact in abnormal genetic and/or molecular prediction.

In this study, radiomic features were selected to construct CR_model for predicting MYCN, 1p and 11q abnormalities, including: PET_wavelet-LLH_glszm_GrayLevelNonUniformity, PET_wavelet-HHH_glszm_SizeZoneNonUniformity, CT_exponential_glrlm_LongRunEmphasis, CT_wavelet-HHL_firstorder_Maximum, PET_squareroot_ngtdm_Contrast, PET_logarithm_firstorder_Minimum, PET_wavelet-LLH_glrlm_LongRunLowGrayLevelEmphasis, PET_wavelet-HHH_glszm_SmallAreaHighGrayLevelEmphasis, PET_wavelet-HHH_glszm_LowGrayLevelZoneEmphasis, CT_exponential_glszm_SmallAreaEmphasis, PET_wavelet-LHL_gldm_DependenceNonUniformityNormalized, CT_wavelet-LLL_glrlm_RunVariance, CT_wavelet-LHL_firstorder_Median, CT_wavelet-LHL_glcm_Imc1, CT_wavelet-HLL_glrlm_LowGrayLevelRunEmphasis, and CT_wavelet-HHH_firstorder_Entropy. The majority of these features (12/16) were not derived from the primary image but from wavelet decomposition images, possibly because wavelet transformed features contained high-order information that may be more helpful for MYCN, 1p and 11q prediction. Previous studies have revealed the potential value of wavelet features in histologic subtype prediction and prognostic assessment (25, 26). In agreement with that, our data also indicated that wavelet features possess remarkable abilities in MYCN, 1p and 11q prediction models. In addition, approximately half of the selected features were extracted from GLRLM (4/16) and GLSZM (5/16). Long run emphasis (LRE) in GLRLM quantifies the distribution of long run lengths, with a larger value representing longer run lengths and more coarse structural textures. Size-zone non-uniformity (SZN) in GLSZM quantifies the variability of size zone volumes in the image, with a smaller value representing more homogeneity in size zone volumes. Our results showed that the greater value of LRE or SZN was correlated with the higher possibility of MYCN amplification and 1p and 11q aberrations.

Currently, ^123^I-MIBG scan is the most frequently used imaging modality and is regarded as standard of care in patients with NB. In comparison with ^18^F-FDG PET/CT, ^123^I-MIBG scan is carried out over 2 days and the image quality is less ideal that could post a challenge to inexperienced physicians (27). At many centers, planar I-MIBG imaging scans are performed, but radiomics based on these images was very limited. Moreover, false- negative MIBG scans were reported as early as 1990, which may result in incorrect down-staging (9). In about 8% of NB patients, false-negative scans at diagnosis occurred despite the solid evidence of disease. ^18^F-FDG PET/CT describes the metabolic state of cancer cells and provides information about malignancy (28). The value of ^18^F-FDG PET/CT in NB has been investigated in many studies. For example, Shulkin et al. demonstrated that ^18^F-FDG uptake was increased in the most of lesions, with about 94% of NB showing elevated ^18^F-FDG activity (28). Melzer et al. reported that ^123^I-MIBG SPECT/CT and ^18^F-FDG PET/CT had significant differences in their uptake patterns. In NB patients, ^18^F-FDG PET/CT had higher sensitivity and specificity for the detection of lesions (9), and showed more extensive primary and/or residual lesions in stage 1 and 2 (8). Overall, ^18^F-FDG PET/CT was superior in depicting NB, although ^123^I-MIBG might be needed to exclude higher-stage (8). Interestingly, the FDG-avid but MIBG-negative and MIBG-avid but FDG-negative NB can coexist in the same tumor (28).

The potential clinical significance of the present study included: (1) radiomics based on pre-therapy ^18^F-FDG PET/CT provides a relatively accurate method in a non-invasive way for predicting MYCN, 1p and 11q, which can be applicable to pediatric NB patients; (2) the status of MYCN, 1p and 11q can be used for risk stratification, therapy selection, therapy response monitor and prognosis prediction.

This study had limitations. Small size cohort from single center may influence the generalized ability, sensitivity and specify of the predictive models. Therefore, prospective larger cohort from multi-center is necessary to validate the results and improve the reliability of models for MYCN, 1p and 11q predictions in NB.

## Conclusion

The models developed by the pre-therapy 18F-FDG PET/CT radiomic signature and clinical parameters are able to predict MYCN amplification and 1p and 11 aberrations in pediatric NB, thus risk stratification, disease management and guiding personalized malignancy therapy in the clinical practice.

## Data Availability Statement

The original contributions presented in the study are included in the article/Supplementary Materials, further inquiries can be directed to the corresponding author/s.

## Ethics Statement

The studies involving human participants were reviewed and approved by Beijing Friendship Hospital, Capital Medical University. Written informed consent from the participants' legal guardian/next of kin was not required to participate in this study in accordance with the national legislation and the institutional requirements.

## Author Contributions

LQ, SY, and SZ made substantial contributions to study design, image acquisition, data analysis and interpretation, and new software creation in this work. SZ, HQ, WW, YK, LL, JL, and HZ contributed writing and/or revising the manuscript. JY and JL approved all versions to be published and were responsible for all aspects of this study. All authors contributed to the article and approved the submitted version.

## Funding

This study was supported by Capital Health Development Research Project (No. 2020-2-2025), National Natural Science Foundation of China (Nos. 81971642, 82001861, and 82102088), and National Key Research and Development Plan (No. 2020YFC0122000).

## Conflict of Interest

LL was employed by the company Sinounion Medical Technology (Beijing) Co., Ltd. The remaining authors declare that the research was conducted in the absence of any commercial or financial relationships that could be construed as a potential conflict of interest.

## Publisher's Note

All claims expressed in this article are solely those of the authors and do not necessarily represent those of their affiliated organizations, or those of the publisher, the editors and the reviewers. Any product that may be evaluated in this article, or claim that may be made by its manufacturer, is not guaranteed or endorsed by the publisher.
