# Supplementary material for: Prediction of MYCN Amplification, 1p and 11q Aberrations in Pediatric Neuroblastoma via Pre-therapy 18F-FDG PET/CT Radiomics
Source: Front Med (Lausanne). 2022 Mar 18;9:840777. doi: 10.3389/fmed.2022.840777 (PMC8971895; doi:10.3389/fmed.2022.840777)
Supplement: Supplementary file 2 [file Table_2.DOCX]

| **Supplementary Table** Comparison of the features and radiomics signature score between positive and negative in both training and test sets | | | | | | | |
| --- | --- | --- | --- | --- | --- | --- | --- |
| feature | training set | | |  | test set | | |
|  | positive | negative | p value |  | positive | negative | p value |
| MYCN |  |  |  |  |  |  |  |
| LDH | 2142.500  (1009.500,3504.500) | 492.000  (339.000,759.750) | <0.0001 |  | 2418.000  (696.500,3149.750) | 328.500  (235.250,536.000) | 0.0001 |
| PET_Original_Min | 0.190  (0.076,0.333) | 0.232  (0.127,0.328) | 0.1907 |  | 0.159  (0.088,0.201) | 0.432  (0.294,0.587) | 0.0005 |
| PET_Gabor_CoD | -5.778  (-7.597,-2.068) | -3.819  (-7.274,-2.295) | 0.4287 |  | -9.655  (-30.641,-8.158) | -2.044  (-3.710,-0.696) | 0.0079 |
| CT_Wavelet_HHH_GLCM_HC | 2085655.685  (1204441.724,  49773565.915) | 3883082.194  (866385.324,  54557440.832) | 0.3575 |  | 1595661.819  (757913.366,  11238276.045) | 1639681.304  (47620.570,  8502769.439) | 0.1928 |
| CT_Wavelet_HHH_GLCM_SIC | 0.100  (0.090,0.109) | 0.084  (0.077,0.097) | 0.0221 |  | 0.098  (0.092,0.107) | 0.096  (0.081,0.109) | 0.2059 |
| CT_Wavelet_LHH_GLCM_Corr | 0.661  (0.611,0.748) | 0.751  (0.699,0.813) | 0.0097 |  | 0.616  (0.598,0.651) | 0.694  (0.655,0.737) | 0.0146 |
| CT_LoG_Skewness | -3.400  (-5.592,-1.183) | -1.807  (-3.149,-0.488) | 0.0339 |  | -4.576  (-5.611,-2.286) | -0.755  (-2.745,0.702) | 0.0207 |
| Rad_score_MYCN | -20.353  (-46.713,-16.016) | -60.859  (-80.381,-44.351) | 0.0004 |  | -2.031  (-14.192,16.020) | -64.383  (-79.129,-42.988) | 0.0001 |
| 1p |  |  |  |  |  |  |  |
| LDH | 1036.500  (582.000,1749.250) | 427.500(339.000,703.500) | 0.0002 |  | 727.000  (560.500,2418.000) | 296.000  (231.000,340.500) | 0.0001 |
| PET_Original_GLSZM_LAHGLE | 432239.447  (73281.743,  596052.537) | 239834.018  (71717.073,  839037.500) | 0.3950 |  | 128544.738  (65969.454,  472991.148) | 82407.353  (2874.200,  169890.996) | 0.0232 |
| PET_Wavelet_HHH_GLSZM_LAHGLE | 9304324.304  (1650253.263,  21025825.222) | 3295647.428  (997959.167,  13075095.125) | 0.0874 |  | 11729396.130  (1963406.834,  16042571.675) | 179857.250  (4910.667,  832671.500) | 0.0004 |
| PET_Wavelet_LHH_GLSZM_SAE | 0.399  (0.283,0.451) | 0.372  (0.299,0.461) | 0.4108 |  | 0.415  (0.364,0.471) | 0.289  (0.215,0.426) | 0.0232 |
| PET_Gabor_CoD | -4.426  (-7.777,-2.520) | -3.743  (-6.687,-2.211) | 0.2468 |  | -9.137  (-17.590,-4.963) | -1.008  (-2.109,-0.496) | 0.0001 |
| CT_Original_NGLDM_DCV | 44.963  (42.041,49.170) | 46.985  (43.817,52.456) | 0.0740 |  | 43.747  (39.451,45.830) | 43.834  (40.942,47.590) | 0.3093 |
| CT_Sobel_GLCM_invVar | 0.458  (0.446,0.472) | 0.439  (0.403,0.457) | 0.0007 |  | 0.457  (0.448,0.466) | 0.428  (0.407,0.442) | 0.0002 |
| CT_Sobel_NGTDM_Strength | 2.394  (1.074,4.087) | 4.986  (1.956,8.667) | 0.0032 |  | 0.511  (0.427,1.397) | 2.642  (0.584,3.814) | 0.1227 |
| CT_Gabor_Kurtosis | 131.688  (50.909,202.006) | 79.888  (36.683,153.000) | 0.0527 |  | 116.281  (44.739,256.322) | 19.107  (5.032,61.363) | 0.0024 |
| Rad_score_1p | -1.168  (-1.999,-0.266) | -2.715  (-4.097,-1.619) | <0.0001 |  | -0.609  (-1.944,0.509) | -2.457  (-3.464,-1.563) | 0.0040 |
| 11q |  |  |  |  |  |  |  |
| LDH | 663.000  (485.000,1065.500) | 387.000  (307.000,1057.000) | 0.0426 |  | 339.000  (242.000,515.000) | 578.000  (296.000,1023.479) | 0.1270 |
| SF | 341.598  (142.700,456.700) | 104.800  (48.400,169.398) | 0.0000 |  | 310.000  (146.200,341.598) | 169.398  (68.600,223.200) | 0.1851 |
| PET_Sobel_Kurtosis | 1.725  (0.683,33.792) | 2.675  (1.260,4.137) | 0.4719 |  | 1.642  (0.661,1.645) | 1.591  (-0.245,3.473) | 0.3904 |
| PET_Sobel_GLCM_SIC | 0.788  (0.759,0.911) | 0.818  (0.764,0.889) | 0.4409 |  | 0.809  (0.786,0.852) | 0.847  (0.784,0.919) | 0.1721 |
| PET_LoG_NGTDM_HDLGE | 4.483  (1.277,10.819) | 5.371  (2.683,14.574) | 0.0426 |  | 11.861  (10.624,19.370) | 5.224  (2.473,25.963) | 0.1721 |
| PET_CoLlAGe_CoD | 0.165  (0.134,1.000) | 0.168  (0.142,0.319) | 0.2615 |  | 1.000  (0.385,1.000) | 0.215  (0.151,1.000) | 0.1480 |
| CT_Wavelet_HHH_Mean | -0.000  (-0.002,0.002) | 0.000  (-0.001,0.003) | 0.1820 |  | -0.002  (-0.002,-0.000) | -0.000  (-0.002,0.002) | 0.1329 |
| CT_Wavelet_HHH_GLCM_SIC | 0.082  (0.075,0.090) | 0.092  (0.082,0.101) | 0.0061 |  | 0.078  (0.077,0.087) | 0.100  (0.088,0.110) | 0.0331 |
| Rad_score-11q | -0.160  (-1.244,0.724) | -1.554  (-2.746,-0.802) | <0.0001 |  | -0.343  (-0.365,1.180) | -2.677  (-4.044,-1.241) | 0.0150 |
| The value of each feature was expressed as median (interquartile range) | | | | | | | |
